# Emerging digital technologies to improve health outcomes in people with intellectual disability: A scoping review

**DOI:** 10.1177/20552076261491906

**Published:** 2026-09-24

**Authors:** Stefan C. Michalski, Tobias Loetscher, Rachael C. Cvejic, Julian N. Trollor

**Affiliations:** 1 National Centre of Excellence in Intellectual Disability Health, Faculty of Medicine & Health, 7800 UNSW Sydney, Sydney, Australia; 2 Cognitive Ageing and Impairment Neurosciences Laboratory, School of Psychology, 695511 Adelaide University, Adelaide, Australia

**Keywords:** intellectual disability, emerging technology, health, digital health, virtual reality, artificial intelligence, healthcare access

## Abstract

**Background:**

People with intellectual disability experience higher rates of multi-morbidity, preventable deaths, and barriers to healthcare access compared to the general population. Emerging digital technologies may address these disparities, but evidence examining their application, effectiveness, and feasibility for people with intellectual disability remains fragmented across multiple disciplines.

**Objective:**

To examine applications of emerging digital technologies for health outcomes in people with intellectual disability across health domains over the past decade.

**Design:**

Scoping review conducted following Joanna Briggs Institute guidelines.

**Method:**

Systematic searches were conducted across PsycINFO, PubMed, and IEEE Xplore in June 2025. Studies were included if they reported on emerging digital technologies (excluding telehealth) supporting health outcomes for people with intellectual disability, presented quantitative or qualitative data, and were published from 2015-2025.

**Results:**

Thirty-six studies were included, spanning seven health domains. Physical activity and motor skills were the most frequently addressed domains, whilst healthcare access and mental health remained underexplored. Technologies included gaming consoles, head-mounted displays, mobile applications, and augmented and mixed reality technology. Most interventions reported improvements in primary outcomes including physical fitness, balance, anxiety reduction, and skill acquisition.

**Conclusion:**

Emerging digital technologies demonstrate feasibility and safety for people with intellectual disability across multiple health domains. To date, research has focused on physical health using consumer technologies. Future research should expand to healthcare access and mental health interventions, involve people with intellectual disability in co-design, and demonstrate pathways to independent use in home and community settings to address documented health disparities.

## 1. Introduction

People with intellectual disability comprise approximately 2% of the adult population,^1,2^ and experience considerable health challenges, including higher rates of multi-morbidity, early onset of frailty, and double the rate of potentially avoidable deaths compared to people without intellectual disability.^3–6^ Healthcare utilisation patterns reflect these disparities, with people with intellectual disability demonstrating increased emergency department presentations and hospital admissions,^7,8^ and a lower uptake of preventative health services.^9–11^ People with intellectual disability face several systemic barriers to equitable healthcare. Health information is often not provided in accessible formats, and communication difficulties can arise when healthcare professionals lack the time, training, or resources to adapt consultations to individual needs. Access is further restricted by workforce shortages, limited specialist services, and poor coordination between health and disability support systems.^12–15^ These barriers can delay care, reduce participation in healthcare decisions, and contribute to poorer health outcomes.^16–18^

Health policy frameworks internationally have identified emerging technologies as one strategy to improve access to care alongside broader systemic reforms.^19–21^ Emerging technologies are relatively novel and rapidly developing technologies that have the potential for substantial impact, but whose future applications and consequences remain uncertain.^22^ Healthcare systems are increasingly adopting digital technologies, evidenced by the expansion of telehealth services and increased patient reliance on digital platforms for health information and service delivery.^23,24^ As these technologies improve and accelerate, people with intellectual disability risk further marginalisation if digital tools are developed without consideration of their needs.^25–27^ Emerging technologies have potential use for receiving healthcare information, accessing services, and supporting clinical interactions. How these tools can be designed and implemented to address rather than exacerbate existing barriers for people with intellectual disability requires evaluation. Technologies designed without accessibility requirements risk creating digital health inequities that amplify rather than reduce existing health disparities.^28,29^

Digital health technologies have demonstrated effectiveness across diverse clinical populations. Virtual reality interventions have reduced symptoms in veterans with post-traumatic stress disorder,^30,31^ whilst digital rehabilitation platforms have improved functional outcomes following stroke.^32,33^ Mobile applications and wearable devices have supported care for older adults living with dementia,^34,35^ children on the autism spectrum,^36,37^ and people with spinal cord injury.^38^ These applications demonstrate that digital tools can be adapted for populations with complex communication, cognitive, and support requirements. People with intellectual disability face persistent healthcare access barriers, yet emerging digital technologies have not been systematically examined for their potential to address these documented disparities.

Recent reviews have focused on established platforms such as telehealth for health assessment^39,40^ and web-based psychological interventions.^41^ Emerging technologies could address barriers across multiple points of healthcare access, including preparing for clinical appointments, supporting communication with healthcare providers, and facilitating understanding of medical information. Applications in healthcare settings require consideration of clinical validity, safety, privacy, and integration within existing care pathways alongside usability.^42,43^ The aim of this scoping review is to examine how emerging digital technologies are being used to address the health needs of people with intellectual disability across all age groups. The review maps the types of technologies applied and summarises evidence about their feasibility and effectiveness across health domains.

## 2. Method

### 2.1. Study Design

This scoping review followed the Joanna Briggs Institute (JBI) methodology for scoping reviews^44^ to explore the use and impact of emerging technologies in addressing the health needs of people with intellectual disability. A scoping review was selected because the aim was to map the breadth of technology applications across health domains rather than to evaluate the effectiveness of a specific intervention for a defined population. Scoping reviews are suited to fields where the primary goal is to identify patterns and gaps rather than synthesise effect estimates.^44^ The review was reported according to the Preferred Reporting Items for Systematic Reviews and Meta-Analyses extension for Scoping Reviews (PRISMA-ScR) checklist to ensure a structured and comprehensive approach.^45^ Consistent with scoping review methodology, no critical appraisal of evidence or risk of bias assessment was conducted.

### 2.2. Eligibility criteria

Articles were included if they met all the following criteria. First, more than half of the study population were people with intellectual disability, or data were separately presented for a subgroup of people with intellectual disability. Second, the article reported findings on the use of an emerging digital technology where people with intellectual disability were the beneficiaries. For this review, emerging digital technologies were defined as interactive, user-facing digital tools that facilitated engagement, learning, communication, or behaviour change, such as virtual reality, mobile health applications, augmented reality, artificial intelligence tools, and serious games. The term “emerging” was applied with reference to novelty within intellectual disability health research rather than novelty in the broader consumer technology landscape. These included extended reality technologies such as virtual reality, augmented reality, and mixed reality; serious games and exergames, which use physical movement, typically captured through motion-sensing or gesture-based hardware, as the primary input for gameplay; artificial intelligence tools such as chatbots; mobile health applications; and accessible digital platforms tailored for people with intellectual disability. Third, the article included an intervention delivered in a health-related setting such as hospitals, clinics, or community health programs, or evaluated an outcome related to health such as physical health, mental health, health literacy, access to health services, supported decision-making in health contexts, or factors influencing health behaviours such as motivation to engage in physical exercise. Fourth, the article presented quantitative or qualitative data. Fifth, the article was written in English. Finally, the article was published between 1 Jan 2015 and 6 June 2025 (date of search). The 10-year window was selected so the review would capture recent developments and applications with relevance to current practice, given the pace of change in consumer and health technology platforms.^23^

Articles were excluded if they met any of the following criteria. First, if the article was a review, meta-analysis, discussion paper, editorial, letter, commentary, or a description of an intervention without outcome data. Second, the intervention involved passive monitoring such as wearable sensors for data collection or did not require active user interaction with the digital technology. Third, the article primarily focused on telehealth.

### 2.3. Search Strategy

A systematic literature search was conducted on 6 June 2025. The following databases were used: PsycINFO, PUBMED, IEEE Xplore. Psychology, Health, and Engineering databases were selected to cover the scope of the current review. The search strategy was designed to include a combination of keywords related to intellectual disability, emerging technologies, and health needs. The full search strategy and complete list of search terms are provided in Supplementary File 1.

### 2.4. Study Selection

Articles were imported into EndNote X9.3.3 (Clarivate Analytics, PA, USA) and duplicates were removed before importing into Covidence (Veritas Health Innovation, Melbourne, Australia) for screening. Two reviewers (SCM, TL) independently screened titles and abstracts. Each full text of potentially relevant articles was then independently reviewed by the same two reviewers (SCM, TL). Any conflicts which arose during both title and abstract screening and full-text screening were resolved through consensus between the two reviewers.

### 2.5. Data Extraction

A standardised data extraction form was created using Microsoft Excel to systematically capture information from the included studies. One reviewer (SCM) performed the primary data extraction, with verification of completeness and accuracy conducted by a second reviewer (TL). Extracted information included publication year, study design, sample size, participant age group, participant involvement in intervention development or delivery, health domain targeted, technology platform, hardware type, interaction mode, implementation setting, support requirements, primary outcome measures, and direction of effect for primary outcomes.

### 2.6. Data analysis and presentation

Descriptive analysis was applied to study characteristics including publication year, location, design, participant demographics, technology platform, and intervention setting to identify patterns across the included studies. Health domains were assigned by reviewing each study’s stated objectives, outcome measures, and intervention focus, and grouping studies by their primary health target, with categories determined inductively from the data rather than using a predetermined framework. For interventions spanning multiple domains, studies were assigned to a single domain based on their primary outcome measures and stated intervention aim. Where multiple outcomes were reported, classification reflected the dominant focus of the intervention rather than all measured outcomes. This approach was preferred over assigning studies to multiple domains, as it preserved the clarity of domain-level comparisons and avoided double-counting in frequency analyses. Technology characteristics were also analysed descriptively, including hardware and software used, interaction methods, and implementation settings. Implementation factors such as support requirements and delivery settings were categorised based on the type and level of assistance described.

Primary outcome data were examined using narrative synthesis. Outcomes were categorised as positive (improvement observed either within a group from pre- to post-test or a reported advantage for the intervention compared with a control or comparison condition), mixed (improvement on some primary outcomes alongside null or inconsistent effects on others), negative (evidence of worsening on primary outcomes or adverse effects), or no change (no meaningful difference observed on primary outcomes from pre- to post-test and no advantage for the intervention). Classification was based on the overall pattern of results rather than statistical significance alone. Findings are presented using frequency distributions, tables, and narrative summaries. Meta-analysis was not conducted due to the diversity of interventions, populations, and outcome measures.

## 3. Results

### 3.1. Study Selection

The systematic search identified 1,313 articles. An additional 6 articles were identified through examining reference lists of included studies. After 68 duplicates were removed, 1,251 articles underwent title and abstract screening. Following title and abstract screening, 65 articles were identified as potentially relevant and moved to the full-text screening stage. After full-text screening, 36 articles met the inclusion criteria and were included in the review. See Figure 1 for the PRISMA flow diagram.

**Figure 1.**
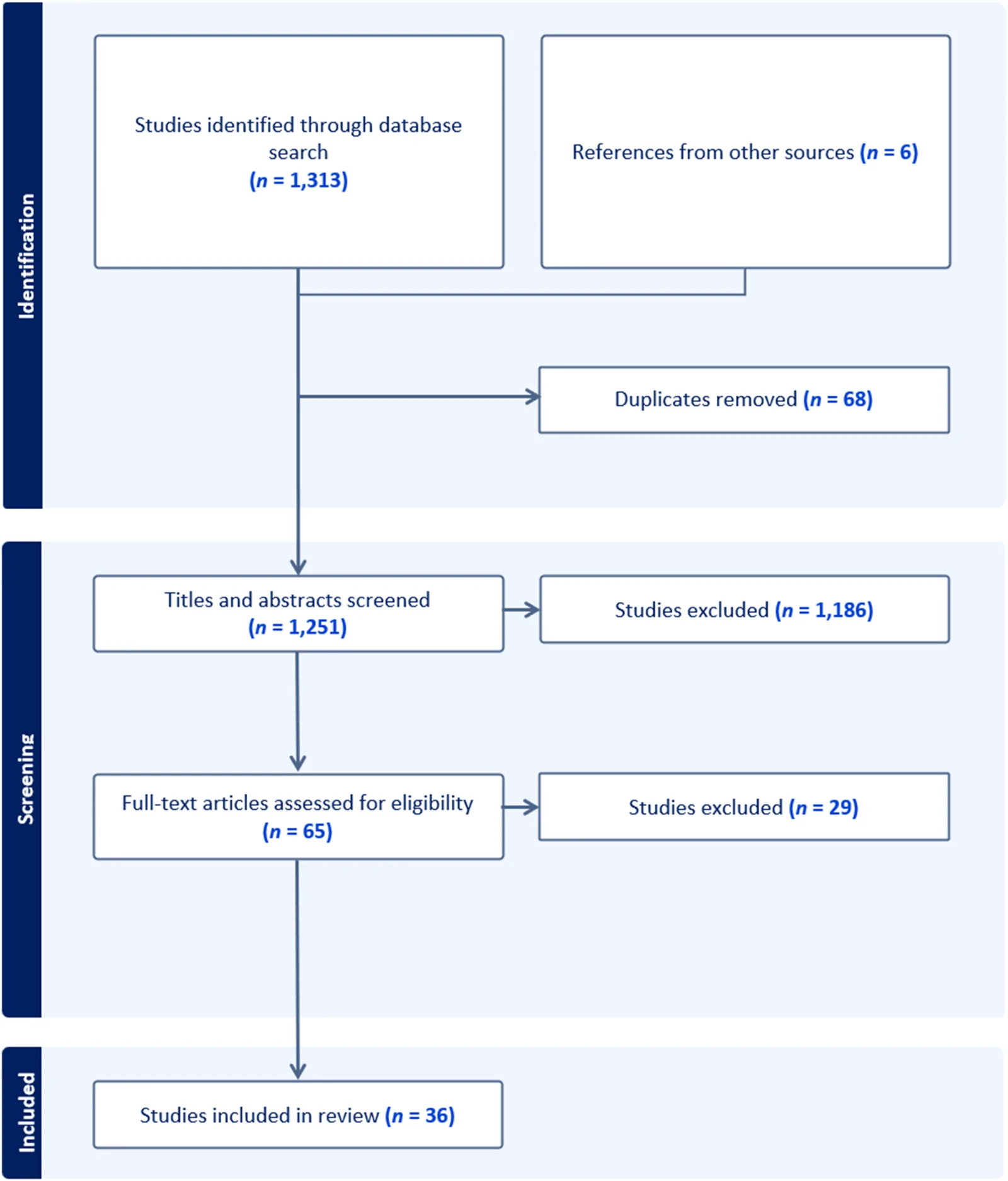
The article selection and screening process using the Preferred Reporting Items for Systematic Reviews and Meta-Analyses (PRISMA) flow diagram.

Of the 36 articles included in this review, publication frequency increased over the past decade (see Figure 2).

**Figure 2.**
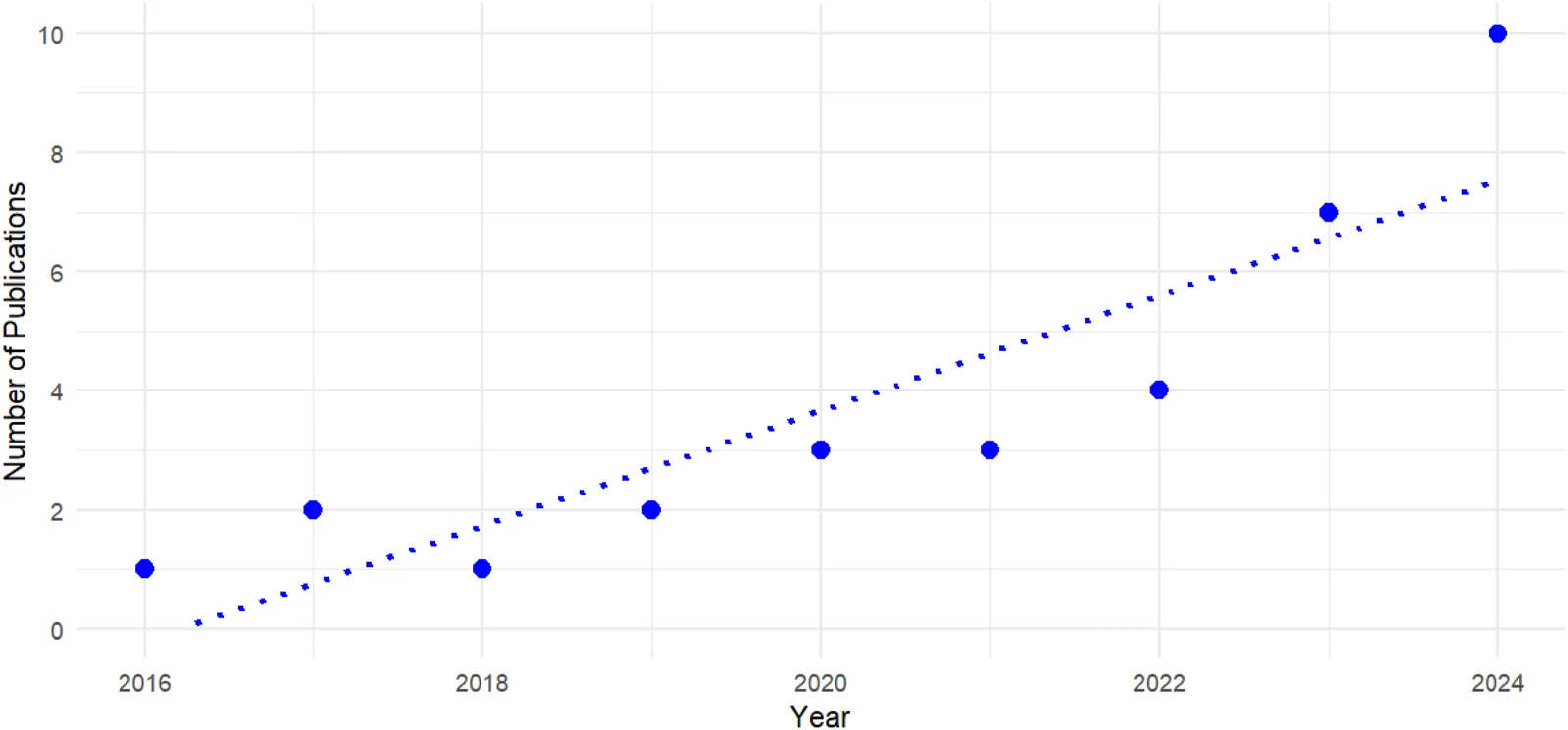
Annual publication frequency of included studies. *Note.* Data for 2025 (*n =* 5) are not shown as the search was completed in June 2025.

### 3.2. Study Characteristics

The review included 36 studies reporting on technology-based interventions involving people with intellectual disability. Most studies were randomised controlled trials (n = 16), followed by single-group pre-post studies (n = 12), non-randomised controlled studies (n = 4), single-case experimental designs (n = 2), and qualitative studies (n = 2). Sample sizes varied (range 3 to 203, median 21), with the largest proportion involving 10–30 participants (*n =* 15). Adults were the most studied group (*n =* 15), with some studies involving children (*n =* 8), adolescents (*n =* 3), or mixed age groups (*n =* 10). Around one-quarter of studies (*n =* 8) involved people with intellectual disability in the development or delivery of interventions. In regard to intervention purpose, physical activity and exercise was the most common intervention purpose (n = 20, 56%), followed by healthcare support (n = 8, 22%), skills training and education (n = 5, 14%), and therapeutic applications (n = 3, 8%). Further details are presented in Table 1. The number of sessions with the technology that each participant completed was not reported in 24 of the 36 included studies. Among the studies that did report it, the number of sessions completed ranged from 2 to 57.

**Table 1.** Characteristics of included studies.

​		n (%)
Study design	Non-randomised controlled study	4
	Qualitative study	2
	Randomised controlled trial	16
	Single-case experimental design	2
	Single-group pre–post study	12
Health issue addressed	Physical activity and fitness	12 (33.3)
	Motor skills, balance and coordination	8 (22.2)
	Mental health and wellbeing	5 (13.9)
	Cognition and communication	4 (11.1)
	Dental and oral health	4 (11.1)
	Nutrition	2 (5.6)
	Healthcare access	1 (2.8)
Sample size	<10	8 (22.2)
	10-30	15 (41.7)
	31-50	7 (19.4)
	51-70	4 (11.1)
	>100	2 (5.6)
Age group of participants	Adults	15 (41.6)
	Adolescents	3 (8.3)
	Children	8 (22.2)
	Mixed	10 (27.8)
Region where the study was conducted	Europe	15 (41.7)
	Asia	11 (30.6)
	North America	5 (13.9)
	Middle East	2 (5.6)
	South America	2 (5.6)
	Oceania	1 (2.8)
Lived experience input	Yes	8 (22.2)
	No or did not specify	28 (77.7)
Intervention purpose	Physical activity and exercise	20 (56)
	Skills training and education	5 (14)
	Healthcare support	8 (22)
	Therapeutic applications	3 (8)
Physical setting	Educational settings	9 (25)
	Care centre/day service	7 (19.4)
	Community/home settings	6 (16.7)
	Healthcare organisation/clinic	5 (13.9)
	Residential care facility	3 (8.3)
	Dental setting	3 (8.3)
	Laboratory/research facility	2 (5.6)
	Rehabilitation/gym	1 (2.8)
Hardware	AR/MR	2 (5.6)
	Computer, phone, or tablet	12 (33.3)
	Game console	13 (36.1)
	Head-mounted display	9 (25)
Support requirements	Active support required	34 (94.4)
	Independent use	2 (5.6)

*Note.* AR/MR = augmented reality/mixed reality.

### 3.3. Health Domains

The 36 included studies spanned seven health domains. Physical activity and fitness formed the most common focus, and motor skills, balance and coordination were also examined often. Fewer studies investigated mental health, cognition and communication, or dental and oral health. Very few studies addressed nutrition or healthcare access. A summary of study characteristics and outcomes is presented in Table 2.

**Table 2.** Details of included studies across health domains.

Health domain	Author	n	Population	Age group	Physical setting	Implementation approach	Hardware	Primary outcomes	Direction of effect
Physical activity and fitness	Henriksen, Martinez-Millana^46^	17	Mild to moderate intellectual disability	Adults	Care centre/day service	Exergaming	Tablet or phone	Engagement with the exergame	Positive
	Lau, Wang^47^	203	Mild intellectual disability	Children	Educational setting	Exergaming	Game console	Body composition; physical activity; motor proficiency	No change
	Lee and Jin^48^	25	Developmental disability (intellectual disability, autism)	Children	Laboratory/research facility	Exergaming	HMD with stationary bike	Physical activity; motor skills; social skills	Positive
	Martinez-Millana, Michalsen^49^	3	Mild to moderate intellectual disability	Adolescents and adults	Care centre/day service	Exergaming	Tablet or phone with stationary bike	Usability; acceptability	Positive
	McMahon, Barrio^50^	4	Intellectual disability and developmental disability	Adolescents and adults	Educational setting	Exergaming	HMD with stationary bike	Duration of exercise; Calories burned; Heart rate	Positive
	Michalsen, Henriksen^51^	9	Moderate intellectual disability	Adolescents and adults	Community/home setting	Physical activity promotion	Phone	Physical activity; Goal Attainment; Social support; Acceptability	Mixed
	Perrot, Maillot^52^	12	Down syndrome	Adults	Healthcare organisation/clinic	Exergaming	Game console	Physical fitness; functional mobility; muscular endurance; cognitive functioning.	Mixed
	Rosa, Tezza^53^	68	Down syndrome	Adolescents and adults	Care centre/day service	Exergaming	Computer or tablet	Heart rate; perceived exertion; motor performance	Mixed
	Ryuh, Chen^54^	7	Mild to moderate intellectual disability	Adolescents and adults	Laboratory/research facility	Exergaming	Game console	Heart rate; perceived exertion; enjoyment	Mixed
	Silva, Campos^55^	27	Down syndrome	Adults	Care centre/day service	Exergaming	Game console	Physical fitness; functional mobility; motor proficiency.	Positive
	Suarez-Villadat, Sadarangani^56^	49	Down syndrome	Adolescents	Educational setting	Exergaming	Game console	Body composition; physical fitness.	Positive
	Suire, Helsel^57^	20	Down syndrome	Adults	Community/home setting	Exergaming	Game console	Attendance; Adherence; Retention; Safety; Exercise intensity	Positive
Motor skills, balance and coordination	Diril, Menek^58^	45	Mild to moderate intellectual disability	Adults	Care centre/day service	Exergaming	Game console	Attention and reaction time; fine motor skills	Positive
	Gómez Álvarez, Venegas Mortecinos^59^	16	Down syndrome	Children	Educational setting	Exergaming	Game console	Motor development; postural control	Positive
	Hsu^60^	24	Mild intellectual disability	Adolescents	Educational setting	Exergaming	Game console	Static balance; dynamic balance; lower limb muscle strength.	Mixed
	Laly, Rosnet^61^	18	Mild to moderate intellectual disability	Adults	Community/home setting	Exergaming	AR/MR	Gait spatiotemporal parameters; cognitive ability.	Positive
	Lee and Jin^62^	23	Developmental disability (intellectual disability, autism, Down syndrome)	Children	Rehabilitation/gym	Exergaming	HMD with stationary bike	Motor skills; physical activity levels	Mixed
	Lotan and Weiss^42^	31	Mild to moderate intellectual and developmental disability	Adults	Residential care facility	Balance training	Game console	Balance	Positive
	Park, Ju^63^	33	Intellectual disability and developmental disability	Children	Educational setting	Exergaming	Game console	Motor coordination; exercise performance	Positive
	Wang, Yu^64^	30	Intellectual disability	Adolescents	Educational setting	Exergaming	Game console	Lower extremity muscle strength; Balance	Mixed
Mental health and wellbeing	Derks, Willemen^65^	159	Mild intellectual disability	Adults	Residential care facility	Serious game training	Computer or tablet	Mentalising	No change
	Geraets, Dolfijn^66^	9	Mild intellectual disability	Adolescents and adults	Healthcare organisation/clinic	Behavioural training	HMD	Well-being; aggression regulation; satisfaction	Positive
	Gosens, Poelen^67^	12	Mild intellectual disability and substance use disorder	Adults	Healthcare organisation/clinic	Motivational interviewing and cognitive-behavioural therapy	Phone	Substance use quantity	Positive
	Gosens, Didden^68^	8	Mild intellectual disability and substance use disorder	Adults	Healthcare organisation/clinic	Motivational interviewing and cognitive-behavioural therapy	Tablet or phone	Usability of the program; perceived treatment effects	Positive
	Mills, Tracey^69^	31	Intellectual disability and autism	Adults	Care centre/day service	Sensory regulation	HMD	Anxiety; Depression; Sensory processing; Personal wellbeing; Adaptive behaviour	Mixed
Cognition and communication	Apriyanti, Mustofa^70^	20	Mild intellectual disability	Children and adolescents	Educational setting	Education	Tablet or phone	Sexual education	Positive
	Costanzo, Fucà^71^	23	Down syndrome	Mixed	Community/home setting	Communication aid	Tablet or phone	Application usability	Positive
	McCormack, Wylie^72^	89	Fragile X syndrome	Adolescents and adults	Community/home setting	Decision support	Tablet	Understanding; Appreciation; Reasoning; Expressing a choice	Mixed
	Torres-Carrión, González-González^73^	6	Down syndrome	Adolescents and adults	Educational setting	Cognitive training	Game console	Cognitive visual-motor abilities	Positive
Dental and oral health	Fakhruddin, El Batawi^74^	22	Down syndrome	Children	Dental setting	Distraction	HMD	Pulse rate; oxygen saturation	Positive
	Jeon, Oh^75^	30	Intellectual disability	Adults	Residential care facility	Skills training	AR/MR	Tooth-brushing performance; oral hygiene status	Positive
	Mehrotra, Shetty^76^	20	Mild intellectual disability	Children	Dental setting	Distraction	HMD	Pulse rate; oxygen saturation; observer-rated anxiety	Positive
	Mehrotra, Manju^77^	40	Mild intellectual disability	Children	Dental setting	Distraction	HMD	Pulse rate; oxygen saturation; observer-rated anxiety	Positive
Nutrition	Janson, Moen^78^	5	Intellectual disability	Adults	Care centre/day service	Education	Tablet	Experiences of using the application	Mixed
	Savage, Candelaria^79^	3	Intellectual disability	Adults	Community/home setting	Education	Tablet or phone	Number of healthy nutrition items identified	Positive
Healthcare access	Acton, Arnold^80^	9	Mild to moderate intellectual disability	Adults	Healthcare organisation/clinic	Desensitisation	HMD	Attendance at a healthcare appointment; fear of attending a healthcare appointment	Positive

*Note.* HMD = head-mounted display. Direction of effect reflects the overall pattern of results for each study’s primary outcomes (positive, mixed, or no change), based on within-group or between-group comparisons, and not statistical significance alone.

#### Physical activity and fitness

Twelve studies used exergaming or other technology-supported activity programmes to address physical activity and fitness in people with intellectual disability. This domain included interventions targeting physical activity levels, cardiovascular fitness, muscular endurance, body composition, and engagement in exercise. Console-based exergaming for children, adolescents, and adults used gesture-based or motion-based interaction and was associated with changes in motor proficiency, motor performance, heart rate responses, enjoyment, adherence, and several components of physical fitness, with some studies also reporting changes in body composition.^52–57^ However, one study reported no change in outcomes for children with mild intellectual disability.^47^ Cycle-based exergaming delivered through a stationary bike and a head-mounted display or tablet/phone was associated with changes in physical activity, motor skills, social skills, usability, acceptability, and exercise duration among children, adolescents, and adults.^48–50^ A motion-based tablet or phone exergame increased engagement among adults with mild to moderate intellectual disability.^46^ A physical activity promotion programme delivered through a mobile phone used touch interaction and an augmented reality game and produced mixed findings for physical activity, social support, and goal attainment.^51^ Across these studies, exergaming and related digital activity programmes increased engagement in physical activity and produced a range of physical, motor, and behavioural outcomes for people with intellectual disability, although several studies reported mixed or no changes.

#### Motor skills, balance and coordination

Eight studies used exergaming and other technology-supported programmes to address motor skills, balance, and coordination in people with intellectual disability. This domain included interventions targeting fine and gross motor skills, static and dynamic balance, postural control, motor coordination, and movement quality including gait and reaction time. Motion-based console exergaming for children, adolescents, and adults was associated with changes in attention, reaction time, fine motor skills, motor development, postural control, static and dynamic balance, lower limb muscle strength, motor coordination, and exercise performance, although some studies reported mixed outcomes for balance measures in adolescents.^58–60,63,64^ Mixed-reality exergaming for adults produced changes in limb muscle strength, gait spatiotemporal parameters, and cognitive ability.^61^ Cycle-based exergaming delivered through a head-mounted display with a stationary bike was associated with changes in motor skills and physical activity levels in children with developmental disability, although findings were mixed.^62^ Balance training delivered through a gesture-based console programme produced improvements in balance for adults with mild to moderate intellectual and developmental disability.^42^ Across these studies, exergaming and related digital activity programmes produced changes in motor skills, balance, and coordination for people with intellectual disability, although several studies reported mixed outcomes.

#### Mental health and wellbeing

Five studies investigated mental health and wellbeing in people with intellectual disability. This domain included interventions targeting psychological and emotional outcomes such as anxiety, depression, stress regulation, self-compassion, and substance use. These interventions used serious games and virtual reality as alternatives to therapies that rely on verbal communication. A serious game application helped adults with borderline to mild intellectual disability reduce stress from negative interpersonal interactions but did not affect their ability to understand their own and others’ thoughts and feelings.^65^ A virtual reality aggression-prevention programme for people with mild to borderline intellectual disability was feasible and acceptable, with high session satisfaction and improved wellbeing scores over the training period. Participants reported learning coping strategies and gaining insight into emotions and aggression triggers; however, three of the nine participants withdrew, and reliable conclusions about changes in aggression could not be drawn.^66^ An immersive virtual reality sensory room improved anxiety, depression, and sensory processing for adults with intellectual disability, but did not change their personal wellbeing or performance across adaptive skill domains including communication, community use, home living, and self-care.^69^ A personality-targeted motivational interviewing and cognitive-behavioural programme supported by a mobile health application reduced self-reported substance use among people with mild intellectual disability or borderline intellectual functioning and substance use disorder and was rated as usable and supportive by participants and their confidants.^67,68^

#### Cognition and communication

Four studies investigated cognition and communication in people with intellectual disability. This domain included interventions targeting cognitive abilities such as reasoning, working memory, and visual-motor skills, as well as communication skills including language development and expression of needs. These interventions addressed communication, working memory and reasoning through decision-making support applications, AI-based assistive technology, and cognitive training platforms. In a sample of people with fragile X syndrome, a computer-based decision aid was associated with higher understanding among participants with higher cognitive functioning. No effects were observed in the full sample.^72^ An interactive digital application increased correct identification of unsafe situations and appropriate responses among children and adolescents with mild intellectual disability in a school-based study.^70^ An artificial intelligence speech-translation application designed to recognise unintelligible speech had good usability and high satisfaction ratings among caregivers of children and young people with Down syndrome.^71^ Training with an active gestural-interaction gaming platform designed for physical and cognitive rehabilitation improved visual–motor coordination, attention, and reasoning in people with Down syndrome, although these effects were not statistically significant when compared to usual classroom activities.^73^

#### Dental and oral health

Four studies investigated dental and oral health in people with intellectual disability. This domain included interventions targeting dental anxiety, treatment engagement during dental procedures, and oral hygiene skills and outcomes. These interventions addressed anxiety, barriers to treatment engagement, and oral health maintenance through virtual reality distraction and augmented reality skill training. Two studies found that audio and virtual reality distraction during dental treatment reduced physiological and behavioural signs of anxiety in children with mild intellectual disability and children without intellectual disability.^76^ Furthermore, audiovisual distraction using video eyewear, combined with computer-controlled local anaesthetic delivery, reduced physiological stress responses and improved treatment engagement during dental procedures for children with Down syndrome.^74^ Augmented reality toothbrush training led to greater improvements in brushing performance and oral hygiene than conventional visual instruction among people with intellectual disability.^75^

#### Nutrition

Two studies investigated digital interventions to improve nutrition among people with intellectual disability. This domain included interventions targeting nutrition knowledge, recognition of healthy food and drink options, and participation in nutrition-related conversations and decisions. These interventions addressed obesity and diet-related behaviours through mobile and tablet-based applications that provided accessible nutrition education and self-management support. A mobile nutrition application with goal-setting and self-monitoring strategies increased recognition of healthy food and drink options, with a medium effect size.^79^ In supervised independent residences, a nutrition application facilitated and normalised nutritional conversations between residents and caregivers, helping residents participate more actively in discussions about their food choices and care.^78^

#### Healthcare access

One study investigated healthcare access for people with intellectual disability. This domain included interventions targeting barriers to attending healthcare appointments, anticipatory anxiety related to unfamiliar healthcare environments and meeting new healthcare professionals. A co-designed VR programme enabled people with intellectual disability to view their specific hospital environment and view personalised recordings from staff.^80^ The videos included short introductions from healthcare professionals to build rapport and reduce anticipatory anxiety. Participants reported attending their scheduled healthcare appointments after completing the intervention and described reductions in fear and improvements in quality of life. Participants also reported positive experiences with the technology, although some encountered technical issues such as motion sickness and headset discomfort.

### 3.4. Technology and Implementation

The technologies used across studies varied in hardware configuration, interaction method, and implementation setting. Gaming consoles were the most common hardware platform (n = 13; 36%) and were used primarily for exergaming interventions involving Nintendo Wii, Xbox Kinect, and Nintendo Switch systems. Tablets, mobile phones, and computers accounted for 12 studies (33%) and were used for mobile-health applications, educational programmes, and communication support. Head-mounted displays were the next most common platform (n = 9; 25%) and were used in virtual and mixed-reality interventions including distraction during dental procedures and immersive preparation for healthcare appointments, with three of those studies using head-mounted displays combined with stationary bikes. Augmented and mixed-reality devices (n = 2; 6%), including Microsoft HoloLens, supported mixed-reality interaction.

Interaction methods differed by hardware platform and intervention purpose. Motion-based interaction (n = 8; 22%) enabled users to control applications through full-body movements and was most often used in console-based exergaming. Gesture-based interaction (n = 7; 19%) used sensor technologies such as Kinect and Leap Motion to detect hand and arm movements. Touch interaction (n = 6; 17%) occurred in tablet and mobile phone interfaces. Cycle-based interaction (n = 4; 11%) appeared in fitness interventions that integrated stationary bikes with either a virtual reality display or a tablet interface. Passive viewing or listening (n = 3; 8%) was used in dental distraction interventions delivered through head-mounted displays. Other interaction modes (n = 8; 22%) included voice interaction for communication aids, mixed-reality interaction, controller-based navigation, augmented reality interaction, and game-based interaction.

Interventions were most frequently delivered in residential and disability service settings (n = 11; 31%), followed by educational settings (n = 9; 25%), healthcare facilities (n = 8; 22%), and community or home settings (n = 6; 16.7%). Most interventions required active support from staff, families, or facilitators (n = 34; 94%), reflecting the support needs of participating individuals and the operational demands of the technologies used.

### 3.5. Efficacy

Based on primary outcomes reported across the included studies, 24 studies (67%) demonstrated positive effects and 12 studies (33%) reported mixed or no change outcomes. No studies reported negative primary outcomes. Interventions using head-mounted displays, AR/MR, and gaming consoles demonstrated higher proportions of positive outcomes than interventions delivered through tablets, mobile phones, or computers. Motion-based, cycle-based, and gesture-based interaction methods were associated with positive outcomes in physical activity and motor skill domains, whereas touch-based and voice-based interactions produced variable outcomes in cognition, communication, and mental health domains. To facilitate comparison across technologies and health domains, Table 3 summarises the number of studies reporting positive primary outcomes only, presented as the number of studies with positive effects relative to the total number of studies examining each domain and technology type.

**Table 3.** Positive outcomes across technology type and health domains.

​	Physical activity and fitness	Motor skills, balance and coordination	Mental health and wellbeing	Cognition and communication	Dental and oral health	Nutrition	Healthcare access	Total
AR/MR	-	1/1	-	-	1/1	-	-	2/2
Game console	3/6	4/6	-	1/1	-	-	-	8/13
HMD	2/2	0/1	1/2	-	3/3	-	1/1	7/9
Tablet, phone or computer	2/4	-	2/3	2/3	-	1/2	-	7/12
Total	7/12	5/8	3/5	3/4	4/4	1/2	1/1	24/36

*Note.* Studies with positive primary outcomes were coded as 1; mixed or no change as 0. Values are presented as number of studies with positive outcomes/total number of studies examining that domain. Totals represent the count of positive outcomes per section. AR/MR = augmented reality/mixed reality; HMD = head-mounted display.

## 4. Discussion

This scoping review examined applications of emerging digital technologies to address health needs of people with intellectual disability over the past decade. Thirty-six studies spanning seven health domains examined the use of emerging digital technologies to support health outcomes for people with intellectual disability, with most studies reporting positive outcomes on primary measures. Most interventions reported positive outcomes, including improved cardiovascular fitness and exercise engagement, enhanced motor coordination and postural control, reduced anxiety and depression, improved reasoning and communication abilities, and better oral hygiene skills. Minor adverse effects such as motion sickness and equipment discomfort were noted in some studies. Overall, these findings should be interpreted as feasibility signals rather than evidence of established efficacy. Research was concentrated in physical activity and motor skills domains, with healthcare access addressed in only one study despite being a well-documented source of health disparities for people with intellectual disability. Gaming consoles were the most common hardware platform, and a substantial proportion of studies used motion-sensing systems that have since been discontinued. Several implementation limitations were also identified. Few studies involved people with intellectual disability in intervention development, most interventions required continuous support during use, and most were delivered in educational, disability service, or clinical settings rather than home or community contexts.

Most studies focused on physical activity and fitness, and on motor skills, balance, and coordination. Across these domains, digital technologies were associated with increased motivation, engagement, and enjoyment of physical activity for people with intellectual disability. Collectively, this body of work provides feasibility evidence for the use of emerging technologies to address selected health needs of people with intellectual disability. In contrast, the distribution of interventions across health domains was uneven. Healthcare access is a well-documented contributor to health disparities for people with intellectual disability,^28^ yet it was addressed in only one included study. Interventions targeting healthcare access typically require co-design with people with lived experience and integration across health and disability service systems.^81–83^ These structural and methodological demands may partly account for the limited representation of healthcare access interventions in the literature.

The reliance on gaming consoles, which accounted for over one-third of included studies, warrants consideration in the context of technology lifecycles. Many studies used Nintendo Wii and Xbox Kinect systems, both of which have since been discontinued. These platforms represented an emerging technology in intellectual disability health research at the time of publication, and their inclusion is consistent with the review’s scope. However, the broader pattern of building evidence around consumer hardware with limited product lifecycles raises questions about translational relevance. The extent to which findings transfer to current hardware depends on the nature of the intervention. Where the core mechanism relies on motion-based interaction, a paradigm that persists in successor systems such as the Nintendo Switch, the underlying principles may translate, even if the specific hardware differs. In these cases, discontinuation of the original platform may be less consequential than it first appears, provided the interaction model is preserved and the evidence base is interpreted at the level of the interaction type rather than the device. By contrast, where findings are tightly coupled to the specific affordances, calibration, or ergonomics of a discontinued system, direct transfer to current hardware is less assured. Service providers seeking to implement these interventions face practical challenges related to hardware availability, maintenance, and replacement costs, and the sustainability of approaches tied to legacy systems in contemporary service contexts remains uncertain.

The review identified methodological limitations in how technologies were developed. Only eight studies involved people with intellectual disability in the design process, with most adopting a model where interventions are developed for people with intellectual disability rather than with them. Technologies developed without direct input from people with intellectual disability may be less customised and less accessible.^83,84^ Co-design approaches, which involve people with intellectual disability as active partners throughout the research process, offer a method for developing interventions that better match user needs and preferences.^81,82,85^ The limited implementation of co-design may reflect several structural barriers. Technology developers may lack training in participatory research methods, leading to co-design being deprioritised in favour of technical development. Small-scale pilot studies, which dominated the current review, may face resource and time constraints that make meaningful co-design difficult to implement. Additionally, challenges persist in ensuring transparent and consistent reporting of how co-design is implemented when it does occur.^86,87^ Future research should establish standards for meaningful participation at all stages of technology development, from problem identification through solution design to outcome evaluation.^88,89^ This requires not only methodological guidance but also adequate funding and timelines that support genuine partnership rather than tokenistic involvement.

The limited involvement of people with intellectual disability in technology development may help explain implementation challenges observed across studies. Most interventions were delivered in institutional or supervised settings, including educational settings, care centres, and healthcare organisations, with only a few studies conducted in community or home contexts. This finding contrasts with assumptions that digital technologies can enable people with intellectual disability to access health interventions without ongoing supervision.^90,91^ The near-universal reliance on facilitated implementation, present in 94% of included studies, has direct implications for scalability. Interventions requiring ongoing staff involvement are more resource-intensive to deliver and less likely to be adopted in settings where workforce capacity is limited.^92^ Current technologies may not be sufficiently accessible or usable to support autonomous use,^93^ and without evidence of independent or minimally supported use, the scalability of these approaches remains unclear. Future research should prioritise developing and testing interventions that people with intellectual disability can use independently in home and community settings.^93–95^

Most studies reported positive outcomes on primary measures, with no serious adverse events reported. These findings indicate that emerging digital technologies are feasible and safe for people with intellectual disability when appropriate support is provided, though they should be interpreted as feasibility signals rather than evidence of established efficacy. Minor adverse effects such as motion sickness and equipment discomfort were addressed through shorter sessions and alternative interaction methods. However, the evidence base has methodological limitations that constrain interpretation of findings. Studies were conducted over short periods in controlled environments which limits the conclusions that can be drawn about safety and effectiveness over time.^96^ The predominance of pilot studies, non-standardised outcome measures, and brief intervention periods prevent conclusions about clinical significance and sustained benefit.^97^ This means the evidence base primarily supports conclusions about acceptability and safety rather than clinical or functional benefit.

Heterogeneity across studies in participant age, cognitive ability, and support needs limited the depth of synthesis achievable within a scoping review framework. The evidence base does not support conclusions about which approaches are most effective for specific age groups, living environments, or support profiles; generating that level of specificity will require larger studies with standardised outcome measures and systematic reporting of participant characteristics and implementation conditions. People with Down syndrome were disproportionately represented, particularly in physical activity and motor skills domains, and findings from these samples may not generalise to people with other aetiologies of intellectual disability. Most research was conducted in high-income countries, limiting understanding of implementation in different resource contexts.^98^ Research conducted exclusively in well-resourced institutional environments limits understanding of how these interventions might perform in home or community settings with variable infrastructure. Positive findings may also reflect publication bias.^88^ Restricting the review to studies published between 2015 and 2025 may have excluded earlier foundational work. The boundary is to some degree arbitrary and was not intended to indicate that a technology remains emerging for a fixed period. The findings therefore reflect recent research rather than the full trajectory of technology use in intellectual disability health.

The review findings indicate several priorities for future research. First, research should move beyond feasibility studies in physical health domains to develop and evaluate interventions that address healthcare access and mental health, where people with intellectual disability face substantial disparities.^28^ Interventions need to be purpose-built to reflect the communication requirements, support needs, and complex health profiles of people with intellectual disability rather than adapting existing models designed for the general population.^99,100^ Digital tools may reduce access barriers through functions such as appointment preparation, communication support with providers, and guided navigation of health systems.^101,102^ However, emerging technologies carry cost barriers related to device acquisition, software licensing, and ongoing technical support that may be prohibitive in lower-resource settings. For people with intellectual disability in these contexts, the gap between technological innovation and accessible implementation may widen rather than narrow existing health disparities. Second, research should establish evidence for sustained benefit beyond the short-term pilot studies that currently dominate the field. Long-term effectiveness studies, cost-effectiveness analyses, and implementation science approaches are needed to determine whether benefits persist, transfer to everyday contexts, and can be sustained within health systems. Third, the expansion of artificial intelligence introduces opportunities including conversational agents and personalised support tools, but accessibility and ethical safeguards must be prioritised from the outset to prevent amplifying existing inequities.^29,103^

## 5. Conclusion

This scoping review found that most studies reported improvements in primary outcome measures for people with intellectual disability across multiple health domains, with few adverse events reported. These findings indicate that emerging digital technologies are feasible for this population, but the evidence base does not yet demonstrate efficacy.Research focused predominantly on physical activity and motor skills. Domains where people with intellectual disability face greater health barriers including healthcare access, mental health, and nutrition remained underexplored. Most interventions required continuous support from staff and were delivered in institutional settings rather than enabling independent use in home and community contexts. Less than one-quarter of studies involved people with intellectual disability in co-design processes, despite evidence that co-design improves the relevance and usability of interventions. Short intervention periods, non-standardised outcome measures, and heterogeneous samples prevent conclusions about long-term effectiveness and which interventions work for which groups. Future research should prioritise interventions for healthcare access and mental health, demonstrate pathways to independent use in home and community settings, and involve people with intellectual disability as partners throughout the research process. Without these shifts, the potential of digital technologies to reduce health disparities for people with intellectual disability is likely to remain unrealised.

## Supplemental material

Supplemental material - Emerging digital technologies for health outcomes in people with intellectual disability: A scoping review Supplemental material for Emerging digital technologies for health outcomes in people with intellectual disability: A scoping review by Stefan C. Michalski, Tobias Loetscher, Rachael C. Cvejic, Julian N. Trollor in DIGITAL HEALTH
