# Supplementary material for: Emerging digital technologies to improve health outcomes in people with intellectual disability: A scoping review
Source: Digit Health. 2026 Sep 24;12:20552076261491906. doi: 10.1177/20552076261491906 (PMC13768656; doi:10.1177/20552076261491906)
Supplement: Supplementary file 1 [file sj-pdf-1-dhj-10.1177_20552076261491906.pdf]

Supplementary File 1

Search strategy

A systematic literature search was conducted across PsycINFO, PubMed, and IEEE Xplore. The same search strategy was applied to all databases.

The following search string was used:

[("intellectual disability" OR "intellectual disabilities" OR "developmental disability" OR "developmental disabilities" OR "learning disability" OR "learning disabilities" OR "special need" OR "special needs" OR "cognitive disability" OR "cognitive disabilities" OR "intellectual developmental disorder" OR "intellectual and developmental disability" OR "intellectual and developmental disabilities" OR "down syndrome" OR "fragile X")

AND

("virtual reality" OR "virtual environment" OR "virtual environments" OR "augmented reality" OR "artificial intelligence" OR "exergame" OR "exergames" OR "virtual game" OR "virtual games" OR "digital health" OR "health technology" OR "health technologies" OR "wearable technology" OR "wearable technologies" OR "interactive technology" OR "interactive technologies" OR "emerging technology" OR "emerging technologies" OR "serious game" OR "serious games" OR "mobile health" OR "mhealth" OR "chatbot" OR "chatbots" OR "mixed reality" OR "AI" OR "XR")

AND

(health OR "mental health" OR "physical health" OR "health literacy" OR "healthcare literacy" OR "health access" OR "healthcare access" OR "decision making" OR anxiety OR depression OR exercise OR fitness OR motivation OR mobility OR rehabilitation OR rehab OR dental OR dentist OR obesity OR weight OR sleep OR wellbeing OR "therapy" OR "mental illness")]
